# Synthesis of a nitrogen doped reduced graphene oxide based ceramic polymer composite nanofiber film for wearable device applications

**DOI:** 10.1038/s41598-022-19234-0

**Published:** 2022-09-16

**Authors:** Jae-Hoon Ji, Gwangseop Lee, Jung-Hyuk Koh

**Affiliations:** 1 School of Electrical and Electronic Engineering, Chung-Ang University, 84 Heukseok-Ro, Dong-Jak Gu, Seoul, 06974 Republic of Korea; 2 Department of Intelligent Energy and Industry, Chung-Ang University, Heukseok-ro, Seoul, 06974 Republic of Korea

**Keywords:** Energy science and technology, Materials science, Nanoscience and technology

## Abstract

In this study, piezoelectric composite nanofiber films were fabricated by introducing nitrogen-doped-reduced-graphene-oxide as a conductive material to a P(VDF-TrFE) polymer and a BiScO_3_–PbTiO_3_ ceramic composite employing an electrospinning process. Nitrogen was doped/substituted into rGO to remove or compensate defects formed during the reduction process. Electro-spinning process was employed to extract piezoelectric composite nanofiber films under self-poling condition. Interdigital electrodes was employed to make planner type energy harvesters to collect electro-mechanical energy applied to the flexible energy harvester. From the piezoelectric composite with interdigital electrode, the effective dielectric permittivity extracted from the conformal mapping method. By introducing BS–PT ceramics and N-rGO conductors to the P(VDF-TrFE) piezoelectric composite nanofiber films, the effective dielectric permittivity was improved from 8.2 to 15.5. This improved effective dielectric constant probably come from the increased electric flux density due to the increased conductivity. Fabricated interdigital electrode using this thin composite nanofiber film was designed and tested for wearable device applications. An external mechanical force of 350 N was applied to the composite nanofiber-based energy harvester with interdigital electrodes at a rate of 0.6 Hz, the peak voltage and current were 13 V and 1.25 μA, respectively. By optimizing the device fabrication, the open-circuit voltage, stored voltage, and generated output power obtained were 12.4 V, 3.78 V, and 6.3 μW, respectively.

## Introduction

Piezoelectric composite materials based on polymers and ceramics have attracted notable attention because of their superior electrical and mechanical properties, such as flexibility, piezoelectricity, and robustness^1–3^. In general, piezoelectric polymers are based mainly on PVDF and P(VDF-TrFE) materials^4,5^. Their electrical properties can be enhanced by adding piezoelectric ceramics to make piezoelectric composite structures. Although piezoelectric composites have been realized, limitations in improving their piezoelectric properties exist owing to their resistive behaviors. To overcome these limitations, conductive materials can be added to piezoelectric composites to improve their electrical properties. Two-dimensional (2D) rGO is widely employed as a conducting material which can be easily mixed with other components to improve electrical and mechanical properties^6–8^. Therefore, introducing rGO into piezoelectric polymers including PVDF and P(VDF-TrFE), can result in improved piezoelectric properties^9,10^. However, many defects are induced during the reduction process of rGO, which can hinder its electron transport properties. These defects can be very detrimental for piezoelectric applications because they disrupt the electric field^11,12^. rGO has been extensively investigated for two dimensional functional device applications owing to its high electrical conductivity and flexibility^13,14^. However, defects originating from the reduction process diminish electrical properties of rGO. To overcome diminished conductive properties, N was doped/substituted into two dimensional rGO. Doping/substitution of N can overcome defects in rGO, resulting in a higher electrical conductivity^6^.

Piezoelectric nanofiber films based on polymer and ceramic ingredients process several advantages compared to other composite structures, such as flexibility and piezoelectricity^13,15^. A nanofiber film has a superior flexibility owing to its high aspect ratio compared to other composite and ceramic materials. An electrospinning process was designed and adopted to fabricate reliable nanofiber and composite nanofiber structures. Electrospinning is a technique that produces nanofibers of polymers, ceramics, and metals by applying an electric field. This process can form nanofibers from complex molecules and can operate at low temperatures^16,17^.

Electrospinning process was employed to fabricate N-rGO doped/substituted piezoelectric composite nanofiber based on P(VDF-TrFE) polymer and BS–PT ceramics. Electrospinning process has many advantages compared with other physical fabrication processes, since it can be low-cost fabrication process by extracting piezoelectric composite nanofiber under self-poling condition. Moreover, highly conductive N-rGO and piezoelectric composite nanofibers can be well mixed during the preparation process before electro-spinning process. As a result, N-rGO doped piezoelectric composite nanofiber can be applied to various different types of wearable device applications.

The main advantage of this synthesized N-rGO with composite nanofiber is that increased conductivity from N-rGO compared with that of rGO. Nitrogen plays role of removing defects in the rGO or substitute with carbon in the rGO materials. Therefore, this increased conductivity can improve floating electrode effects in the piezoelectric composite materials. Also, the representative results of this manuscript compared with other papers can be summarized as follow. N-rGO-doped piezoelectric composite nanofiber composites based on polymer and BiScO_3_–PbTiO_3_ were fabricated in the form of planar piezoelectric energy harvesters with interdigital electrodes. It was the first time to report the improved floating electrode effects based on the planner type piezoelectric energy harvesters.

For the device applications, interdigital electrodes were designed and employed to N-rGO doped/substituted piezoelectric composite nanofiber based on P(VDF-TrFE) polymer and BS–PT ceramics. Almost all wearable devices are based on the planner type structure, classical vertical type electrode cannot be applied for the device applications. Applied mechanical forces can be converted to the electrical energies through the interdigital electrode on the piezoelectric nanofibers. The effective dielectric permittivity can be simulated and calculated by employing the conformal mapping process. By extracting the different values of effective dielectric permittivity of piezoelectric composite nanofibers, we believe that N-rGO doped piezoelectric nanofibers with planner type electrode can be applied for the various wearable device applications.

In this study, composite nanofiber films comprising N-rGO-incorporated P(VDF-TrFE) polymers and BiScO_3_–PbTiO_3_ ceramics were prepared using electrospinning. Flexible piezoelectric energy harvesters based on these composites were investigated for use in wearable electronic applications.

## Experimental procedure

### Preparation of BS–PT piezoelectric ceramic powders

Bi_2_O_3_, Sc_2_O_3_, PbO and TiO_2_ powers were employed as the raw materials. By considering the volatilization degree of Bi_2_O_3_ elements, 0.01 mol excessed Bi was added to BS–PT compositions. The mixture was milled with stabilized zirconia ball. Then, the mixture were calcined and sintered in powder form. The sintered powders were controlled to have less than 50 μm. Finally, the powders were ball-milled by planetary milling.

### Synthesis of rGO and N-rGO powder

Nitrogen doped rGO was prepared by a modified Hummers method^18^. Graphite and NaNO_3_ powder were added to H_2_SO_4_ by stirring in bath. KMnO_4_ was then added slowly to the solution. Deionized water was added to the solutions, and stirred for 1 h, followed by the addition of 10 mL of H_2_O_2_. To eliminate oxidant ions and other inorganic impurities, distilled water and 1:10 HCl aqueous solution were added and centrifuged, and the mixture was washed several times. Hydrazine hydrate was added to the solution to fabricate rGO. To produce nitrogen-doped rGO, graphene oxide and NH_3_NO_3_ were added to ethanol solution and stirred. To remove the ethanol, the solution was heated to 60 °C. The dried mixture was calcined and washed with deionized water and ethanol.

### Fabrication of the nanofiber composite films

**Figure 1:**
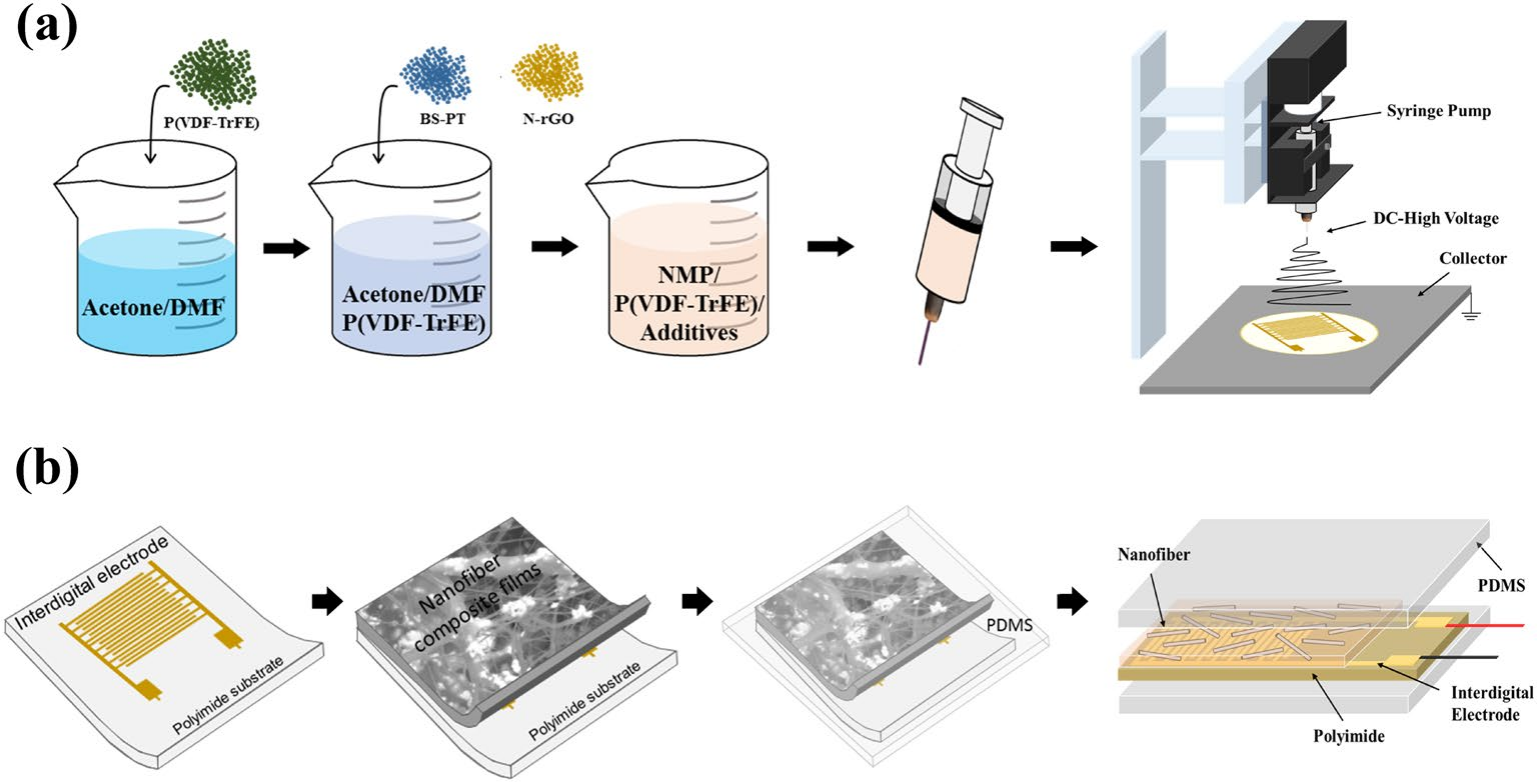
Diagrams of fabrication processes for (**a**) nanofiber composite film and (**b**) piezoelectric energy harvester.

Figure 1a shows a schematic of the fabrication process of a flexible piezoelectric energy harvester based on a composite nanofiber film. To prepare the composite solution, P(VDF-TrFE) (Solvay Co. Ltd.), acetone (Sigma-Aldrich Co. Ltd. , purity: 99.5%), and N, N-dimethylformamide (DMF, Sigma-Aldrich Co., Ltd., purity: 99.8%) were mixed in a weight ratio of 2:5:5 and stirred for 24 h. Then, BiScO_3_–PbTiO_3_ (BS-PT) nanoparticles (30 wt.%) and N-rGO powders (5 wt.%) were added to the mixture and stirred. Finally, P(VDF-TrFE) and P(VDF-TrFE)/BS-PT composite solutions were obtained. The composite solution was loaded into a 10 mL plastic syringe with a 21 G metal nozzle and then electrospun. The electrospinning process was performed using an electric field of 1.2 kV/cm, feed rate of 1 mL/h, distance between the needle tip and the collector of 12 cm, and substrate temperature of 55 °C.

As shown in Fig. 1a, the electrospinning process was applied to a polyimide substrate coated with an interdigital electrode to fabricate an energy harvesting device based on composite nanofiber films. Figure 1b shows a schematic of the fabrication process for a piezoelectric energy harvester with interdigital electrodes. The interdigital electrode had twenty pairs of Cu fingers with sizes and gaps of 100 µm. Interdigital electrodes are effective for measuring planar capacitances; therefore, these electrodes are useful in energy harvesting applications, where a large number of piezoelectric charges are developed and retained. After this process, polydimethylsiloxane (PDMS) was used to encapsulate the device for stability and protection. The crystal structures of the samples were determined via X-ray diffraction (XRD, Bruker-AXS; New D8-Advance). Field-emission scanning electron microscopy (FE-SEM, Carl Zeiss, SIGMA HD) was performed to study microstructures of composite nanofiber films. The generated output voltage and current were analyzed using an oscilloscope (DSO-X2002A, Agilent Technologies) and a Femto/Picoammeter (B2981A, Agilent Technologies). The stored voltage of the energy harvesting device was measured in the external capacitor of a full-bridge rectifier.

## Results and discussion

**Figure 2:**
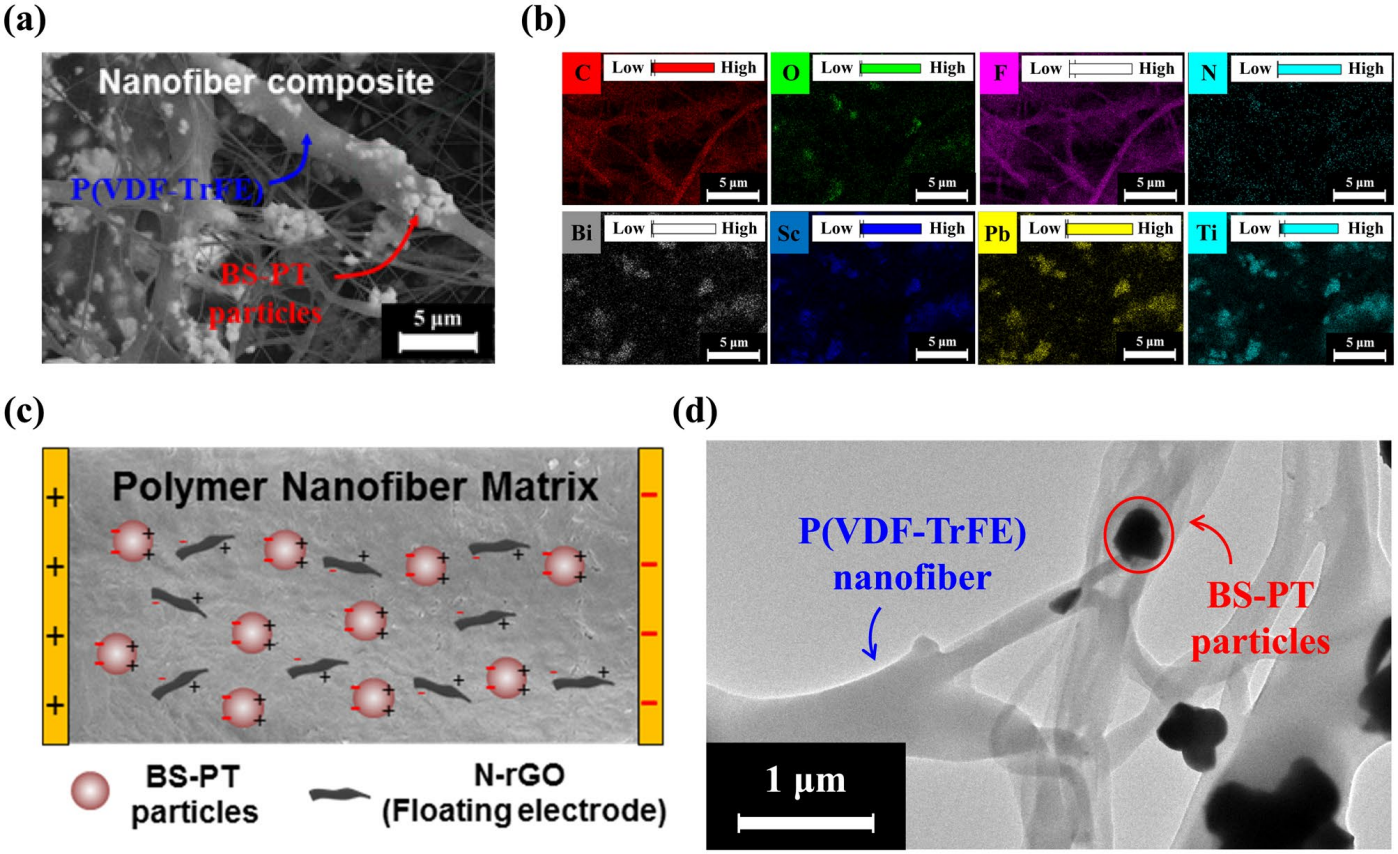
(**a**) FE-SEM image of P(VDF-TrFE)/BS-PT/N-rGO nanofiber composite film, (**b**) EDS data for the P(VDF-TrFE)/BS-PT/N-rGO nanofiber composite film with different composite (**c**) Schematic diagram of floating electrode component of N-rGO in nanofiber structure, (**d**) clear TEM images of TEM images of P(VDF-TrFE)/BS-PT/N-rGO film.

Figure 2a shows FE-SEM image of P(VDF-TrFE)/BS-PT/N-rGO composite nanofiber films. The composite nanofiber was long and smooth, with a diameter of approximately 800 nm. Sub-micrometer particles were well dispersed on the nanofiber, which suggests a high compatibility between sub-micron-sized functional ceramic particles and the N-rGO-doped P(VDF-TrFE) matrix. In the Fig. 2b, EDS compositional analysis data were displayed with different colors. Different elemental compositions were detected. Composition of C, F, N, O, Bi, Sc, Pb, and Ti were distributed in P(VDF-TrFE)/BS-PT/N-rGO composite nanofiber films; the corresponding atomic and weight percentages are listed in Table 1.

**Table 1:** Elemental composition of P(VDF-TrFE)/BS-PT/N-rGO.

Element	C	F	N	O	Bi	Sc	Pb	Ti	Total
Atomic (%)	48.01	28.69	0.42	13.73	2.88	2.98	1.62	1.67	100
Weight (%)	23.07	21.80	0.24	8.79	24.10	5.35	13.44	3.21	100

Figure 2c shows a schematic of floating electrodes of conductive N-rGO particles contained in the composite nanofiber structure. Dispersed conductive N-rGO act as floating electrodes in composites, which help collect charges from piezoelectric materials.

Figure 2d shows the TEM images measured in the mass-thickness contrast mode. In the bright field image mode Fig. 2c, the ceramic parts can be seen in the dark images on the while, the polymer part can be seen in the bright color. Therefore, we expect that piezoelectric BS–PT ceramic particles and conductive N-rGO floating electrodes in composite nanofiber structures will enhance the output power of energy harvesters.

**Figure 3:**
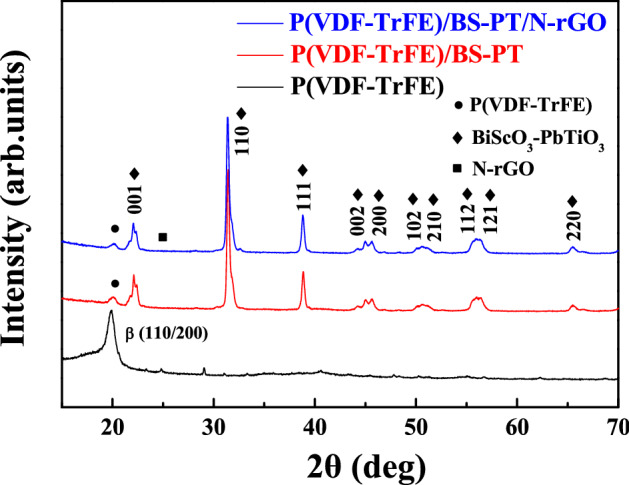
XRD patterns of P(VDF-TrFE), P(VDF-TrFE)/BS-PT, and P(VDF-TrFE)/BS-PT/N-rGO nanofiber films.

X-ray diffraction (XRD) patterns of P(VDF-TrFE), P(VDF-TrFE)/BS–PT, and P(VDF-TrFE)/BS–PT/N-rGO composite nanofiber films is shown in Fig. 3. P(VDF-TrFE) films show peaks of the β phase (110/200) at 2θ = 19.8°. This β-phase polymer structure has ferroelectric properties resulting from the atomic arrangement. XRD patterns of P(VDF-TrFE)/BS–PT composite nanofiber films indicated the formation of the β-phase polymer structure; however, the crystallinity was lower than that of the P(VDF-TrFE)/BS–PT composite film. The amount of the β-phase polymer appeared to decrease during the fabrication process. However, a small amount of the β-phase polymer remained after the processing. BS–PT peaks in XRD measurements indicated that BS–PT piezoelectric nanoparticles were well dispersed in P(VDF-TrFE)/BS–PT composite nanofiber films. This result suggests that BS–PT ceramic nanoparticles were not only effectively dispersed in P(VDF-TrFE) nanofibers but were also undisturbed during the electrospinning process.

**Figure 4:**
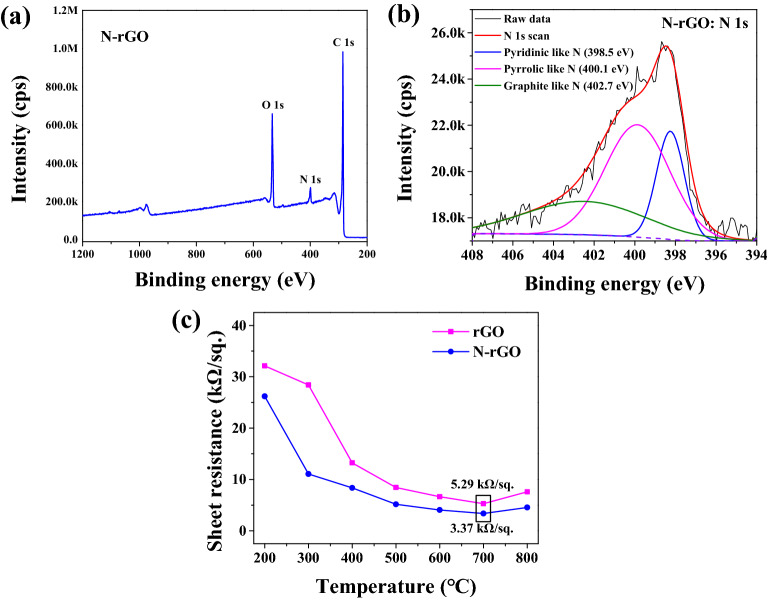
(**a**) XPS survey and (**b**) N 1 s scan of synthesized N-rGO and (**c**) sheet resistance according to rGO, N-rGO deposition temperature.

Figure 4a shows the XPS survey scan spectrum of N-rGO and the chemical composition of each element. Carbon, nitrogen, and oxygen peaks appeared around 285.2 eV, 399.5 eV, and 533.0 eV, and the atomic ratios were confirmed to be 73.9%, 6.1%, and 20.0%, respectively.

Figure 4b shows the bonding state of N 1 s. In N-rGO, the N 1 s peak can be separated into pyridinic-like N (398.5 eV) which represents the nitrogen located on the 6-membered ring, pyrrolic-like N (400.1 eV) which represents the nitrogen located on nitrogen in a five-membered ring, and graphitic-like N (402.7 eV) peaks^19^. Pyridinic-like N, pyrrolic-like N, and graphitic-like N binding ratio were approximately 21.2%, 51.3%, and 27.5%, respectively.

Figure 4c shows sheet resistance of rGO and N-rGO films after the rapid thermal annealing (RTA) process. As a result of performing the RTA process at 700 °C, the sheet resistance the lowest value of the rGO film was 5.29 kΩ/sq. The N-rGO film was 3.37 kΩ/sq. At all processing temperatures, the sheet resistance of the N-rGO film was lower than that of the rGO film. Therefore, due to the high conductivity of N-rGO, the N-rGO-based piezoelectric composite nanofiber films are expected to have improved electrical properties and piezoelectricity.

**Figure 5:**
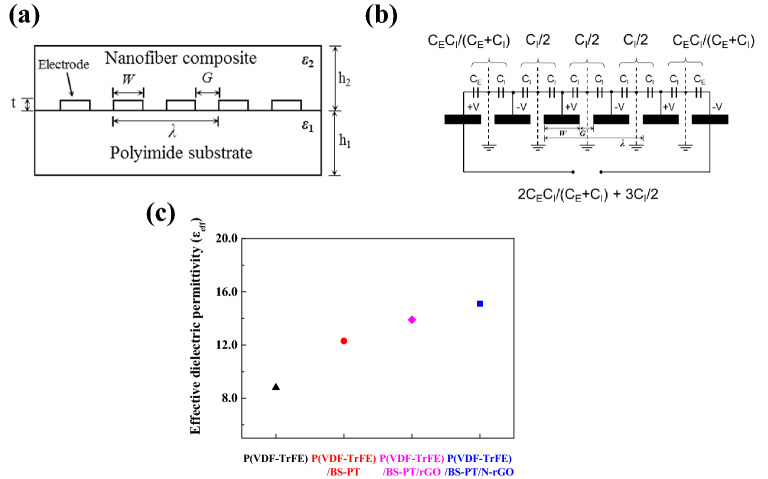
(**a**) Cross-section view of two-layer substrate for IDC, (**b**) equivalent circuit for IDC, and (**c**) effective dielectric permittivity of composite nanofiber films.

Figure 5a shows a schematic of a flexible PI-substrate-based composite nanofiber energy harvester with interdigital electrodes. A conformal mapping technique was applied to extract the effective dielectric permittivity from the analyzed capacitance of the interdigital electrode. Conformal mapping analysis allows the electric field distribution to be changed from rectangular to circular coordinates. As a result, effective dielectric permittivity of the composite nanofiber film and the PI substrate, *ε*_n_ and *ε*_s_, could be estimated, as shown in Fig. 5a. The indispensable condition for using a conformal mapping to a two-layered substrate is *ε*_n_ > *ε*_s_. Otherwise, the electric field cannot be confined within the composite nanofiber layer. As shown in Fig. 5a, W is the finger width, G is the space between fingers, λ is the spatial wavelength of the interdigital capacitor (IDC), t is the thickness of the metal electrode pattern, and h_n_ is the height of the nanofiber film and substrate. We obtained analytical models of the IDC following the work of Gevorgian^20,21^. Modified Igreja’s equations for capacitances of inner (C_I_) and outer (C_E_) electrodes were determined, as shown in Fig. 5b, where it was assumed that the substrate thickness was non-infinite and the air layer below the substrate was infinitely thick. Equations of the IDC can then be expressed as follows^21^:

1 $$ C_{IDC} = \left( {N - 3} \right)\frac{{C_{I} }}{2} + 2\frac{{C_{I} C_{E} }}{{C_{I} + C_{E} }}, $$

2 $$ \begin{aligned} C_{I} = & 2C_{I,air} + C_{I,n} + C_{I,s} \\ = & \varepsilon_{0} L(2\frac{{K\left( {k_{I\infty } } \right)}}{{K\left( {k_{I\infty }^{^{\prime}} } \right)}} + \left( {\varepsilon_{n} - 1} \right)\frac{{K\left( {k_{I,n} } \right)}}{{K\left( {k_{I,n}^{^{\prime}} } \right)}} + \left( {\varepsilon_{s} - 1} \right)\frac{{K\left( {k_{I,s} } \right)}}{{K\left( {k_{I,s}^{^{\prime}} } \right)}}, \\ \end{aligned} $$

3 $$ \begin{aligned} C_{E} = & 2C_{E,air} + C_{E,n} + C_{E,s} \\ = & \varepsilon_{0} L(2\frac{{K\left( {k_{E\infty } } \right)}}{{K\left( {k_{E\infty }^{^{\prime}} } \right)}} + \left( {\varepsilon_{n} - 1} \right)\frac{{K\left( {k_{E,n} } \right)}}{{K\left( {k_{E,n}^{^{\prime}} } \right)}} + \left( {\varepsilon_{s} - 1} \right)\frac{{K\left( {k_{E,s} } \right)}}{{K\left( {k_{E,s}^{^{\prime}} } \right)}}, \\ \end{aligned} $$

where K is elliptical integrals defined bellow; k and k′ are argument of each elliptical integral defined below; ${C}_{I,n}$, ${C}_{I,s}$, ${C}_{E,n}$, and ${C}_{E,s}$ are interior and exterior electrode capacitances of the nanofiber film (n) and the substrate (s), respectively; *L* is the length of fingers; *ε*_n_ is the effective dielectric permittivity of the nanofiber film; and *ε*_s_ is the effective dielectric permittivity of the substrate. In addition, the relationship between moduli of elliptical integrals *k* and *k'* are as follows:

4 $$ k^{\prime} = \sqrt {\left( {1 - k^{2} } \right)} . $$

This conformal mapping analysis allowed for the effective dielectric permittivity to be calculated. The extracted dielectric constant, as shown Fig. 5c, was approximately 8.2, 12.3, 13.9 and 15.5 for P(VDF-TrFE), P(VDF-TrFE)/BS–PT, P(VDF-TrFE)/BS–PT/rGO and P(VDF-TrFE)/BS–PT/N-rGO, respectively. The higher effective dielectric permittivity of P(VDF-TrFE)/BS–PT/N-rGO was attributed to the floating electrode behavior, as described in Fig. 2b. The floating N-rGO electrode in the composite nanofiber causes charges to be more easily attracted and collected by interdigital electrodes. Therefore, the effective dielectric permittivity was improved owing to increased electric flux densities resulting from the increased charge in interdigital electrodes. In addition, due to the high conductivity of N-rGO, it can be seen that the formation of charges in the composite film is strengthened and the dielectric properties are improved compared to that of rGO.

**Figure 6:**
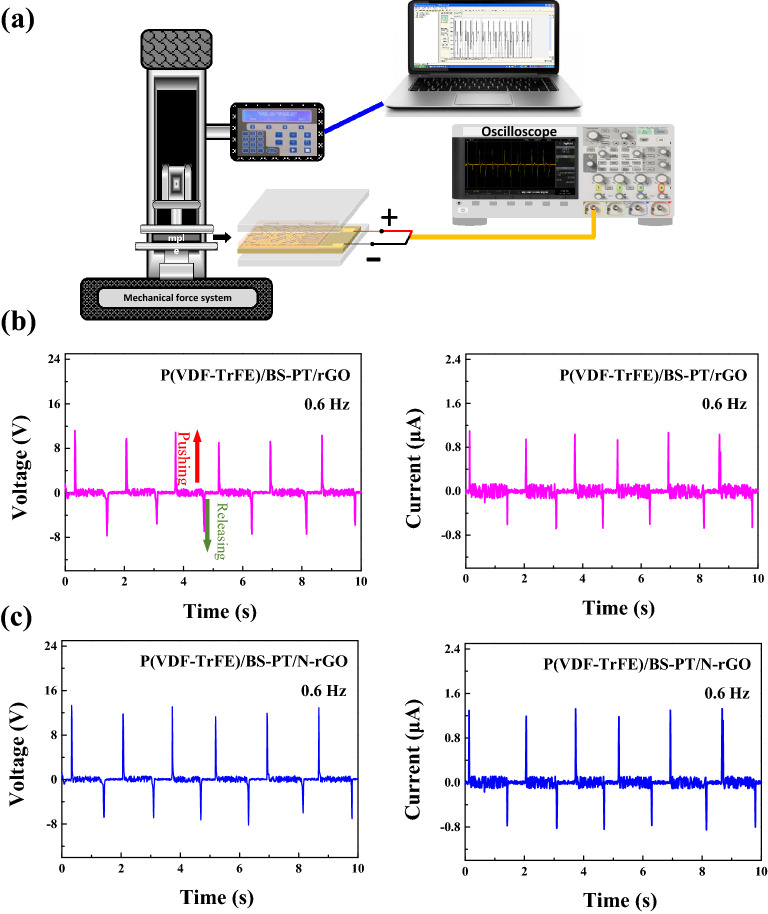
(**a**) Schematic of measurement system, and open-circuit voltage and short-circuit current of (**b**) P(VDF-TrFE)/BS-PT/rGO, (**c**) P(VDF-TrFE)/BS-PT/N-rGO energy harvester based on composite nanofiber films.

Figure 6a shows a schematic of the measurement system for the energy harvester. The mechanical force system was connected to an energy harvester with a circuit system and was controlled by a computer. An external mechanical force of 350 N was applied to the composite nanofiber with interdigital electrodes at a rate of 0.6 Hz. The generated output power was recorded by the computer. Figure 6b,c show repeated positive and negative output voltages and currents generated during the continuous pushing and release of the external mechanical force for P(VDF-TrFE)/BS–PT/rGO, P(VDF-TrFE)/BS–PT/N-rGO. The generated open-circuit voltage and short-circuit current were measured and recorded. The peak voltage and current of the energy harvester based on the composite nanofiber film were 11.2 V and 1.09 μA in P(VDF-TrFE)/BS–PT/rGO, 13 V and 1.25 μA in P(VDF-TrFE)/BS–PT/N-rGO. Positive values of voltages and currents are attributed to the applied stress, while negative values are attributed to the release of stress; hence, negative values are always smaller in magnitude than positive values.

**Figure 7:**
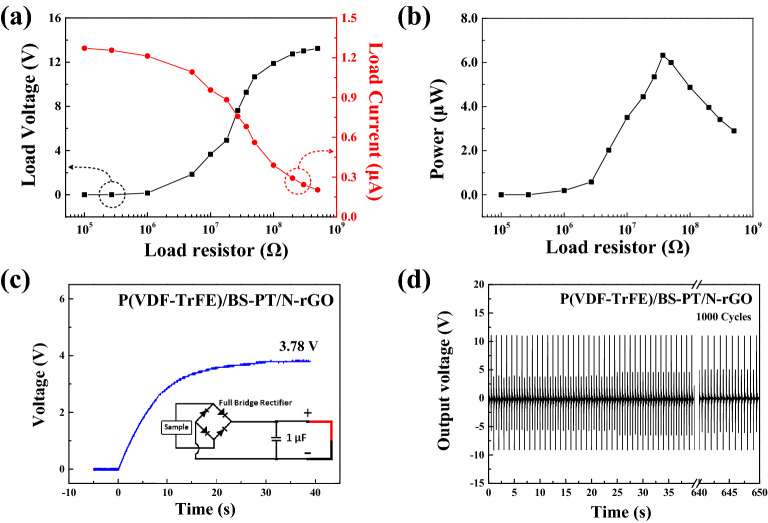
(**a**) Output voltage and current, (**b**) power, (**c**) stored voltage, and (**d**) reliability of nanofiber composite film energy harvester.

Figure 7a shows measured output voltages and currents of the energy harvester based on the composite nanofiber film with different loading resistances. To measure the generated output power of the energy harvesting system, a load resistor or capacitor was used to measure the maximum output power and energy. The maximum power density was obtained by optimizing the load resistance. By varying the load resistance, the output load current was decreased from 1.27 to 0.2 μA, and the output load voltage was increased from 0.15 to 13.2 V.

Figure 7b shows the output power generated by the energy harvester. The output power was calculated from the voltage and current across the load. The output voltage and current were measured at an external load resistance, ranging from 100 kΩ to 500 MΩ, that was connected to the composite nanofiber film energy harvester. The output power can be expressed as:

5 $$ P = I_{L} V_{L} , $$

where *I*_*L*_ and *V*_*L*_ are the output current and voltage across the load resistance, respectively. As shown in Fig. 7b, the output power of the energy harvester first increased and then decreased. The maximum output power was 6.3 μW at an optimized load resistance of 37 MΩ, corresponding to a voltage of 9.27 V and a current of 0.68 μA. After this peak value, the generated output power decreased. Additionally, the power density can be expressed as:

6 $$ Power Density = \frac{{\text{Generated output power}}}{volume}. $$

The generated output power of the piezoelectric energy harvester based on the composite nanofiber film was 0.63 mW/cm^3^.

The load current can be expressed as:

7 $$ I_{L} = \frac{V}{{R_{piezo} + R_{L} }}, $$

where *R*_*piezo*_ and *R*_*L*_ are resistances of the composite nanofiber film and the load, respectively. Therefore, Eq. (5) can be expressed as:

8 $$ P_{L} = I^{2} R_{L} = \left( {\frac{V}{{R_{piezo} + R_{L} }}} \right)^{2} R_{L} = \frac{{V^{2} }}{{R_{piezo}^{2} /R_{L} + 2R_{piezo} + R_{L} }}. $$

The maximum value of *P*_*L*_ occurs at the minimum value of the denominator, and therefore, the derivative of the denominator of *P*_*L*_ can be expressed as:

9 $$ \frac{d}{{dR_{L} }}\left( {R_{piezo}^{2} /R_{L} + 2R_{piezo} + R_{L} } \right) = - \frac{{R_{piezo}^{2} }}{{R_{L}^{2} }} + 1 = 0. $$

Consequently, the maximum P_L_ value occurs when *R*_*piezo*_ = *R*_*L*_. In our study, the optimized load resistance was measured to be 37 MΩ, and therefore, according to the above formula, the resistance of the piezoelectric energy harvester, ${R}_{piezo}$, is estimated to be 37 MΩ.

Figures 7c,d show the stored voltage and reliability results of the energy harvester based on the composite nanofiber film. The stored voltage of the energy harvester increased up to 3.78 V when mechanical forces were applied. The output properties of composite nanofiber film were compared with other polymer/ceramic piezoelectric composites and are summarized in Table 2^22–26^. As shown in the Table 2, compared with other researches, highly conductive N-rGO was introduced to piezoelectric composite nanofiber to improve electro-mechanical properties, which employed in the energy harvester. Therefore, the output energy from the energy harvester was increased by large margin compared with other recent result. The result was compared and listed in the Table 2.

**Table 2:** Comparison of electrical properties of flexible piezoelectric composite films on different piezoelectric materials.

Piezoelectric materials	Output voltage	Power	Characteristics	Reference
KNN/P(VDF-TrFE)	170 mV	4 nW	Spin coating film	^22^
PZT/P(VDF-TrFE)	2 V	–	Electrospun film	^23^
m-PZT/P(VDF-TrFE)	3.4 V	–	Electrospun film	^23^
PVDF/rGO/MoS_2_	2.4 V	0.81 μW	Thin film	^24^
P(VDF-TrFE)/BTO/rGO	4.65 V	–	Spin coating film	^25^
P(VDF-TrFE)/ PMN-PT/rGO	8.4 V	–	Thin film	^26^
P(VDF-TrFE)/BS-PT/N-rGO	12.4 V	1.25 μW	Electrospun film	This work

Periodic external mechanical forces were applied to examine the reliability of the output performance of the composite nanofiber energy harvesting system. More than 1000 cycles of mechanical forces were applied to the composite-nanofiber-based energy harvester. The applied mechanical force was approximately 300 N at a rate of 1.5 Hz. The generated voltage was recorded simultaneously using an oscilloscope. The flexible piezoelectric energy harvester exhibited a stable output performance even under a constant external pressure. The results indicate that the energy harvester based on the composite nanofiber film exhibits an outstanding output performance without any fatigue.

**Figure 8:**
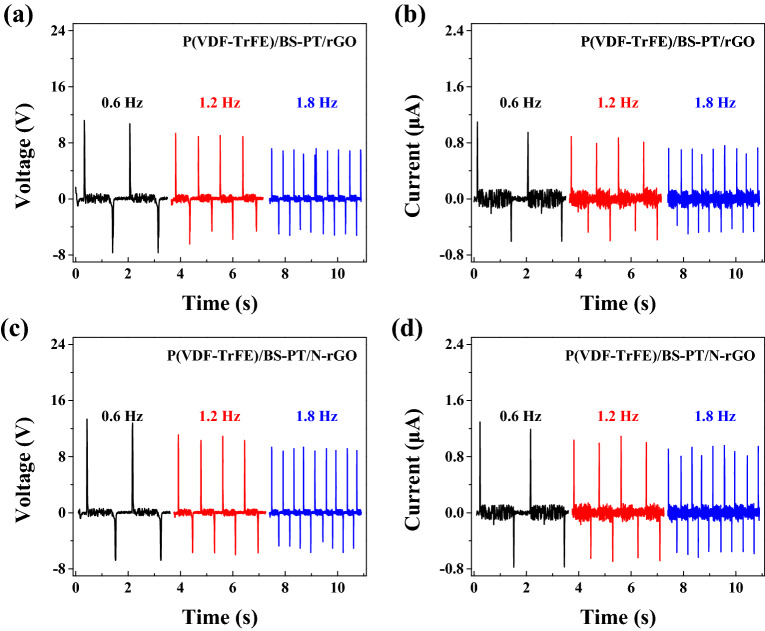
Open circuit voltage and short circuit current at 0.6, 1.2 and 1.8 Hz for (**a**), (**b**) P(VDF-TrFE)/BS-PT/rGO and (**c**), (**d**) P(VDF-TrFE)/BS-PT/N-rGO piezoelectric composite nanofiber films.

Figure 8 shows output performance of wearable devices under different loading frequencies in P(VDF-TrFE)/BS–PT/rGO and P(VDF-TrFE)/BS–PT/N-rGO composite nanofiber films. As increasing the loading frequency, the output voltages and current were decreased. In P(VDF-TrFE)/BS–PT/rGO, the output voltages and current were decreased from 11.2 V, 1.09 μA to 7.2 V, 0.72 μA, and in P(VDF-TrFE)/BS–PT/N-rGO composite nanofiber film, it decreased from 13.3 V and 1.29 μA to 9.3 V and 0.91 μA. P(VDF-TrFE)/BS–PT/N-rGO showed better output performance than P(VDF-TrFE)/BS-PT/rGO at all loading frequencies. As a result, after stress relief, stress was applied before recovery, resulting in reduced output performance.

**Figure 9:**
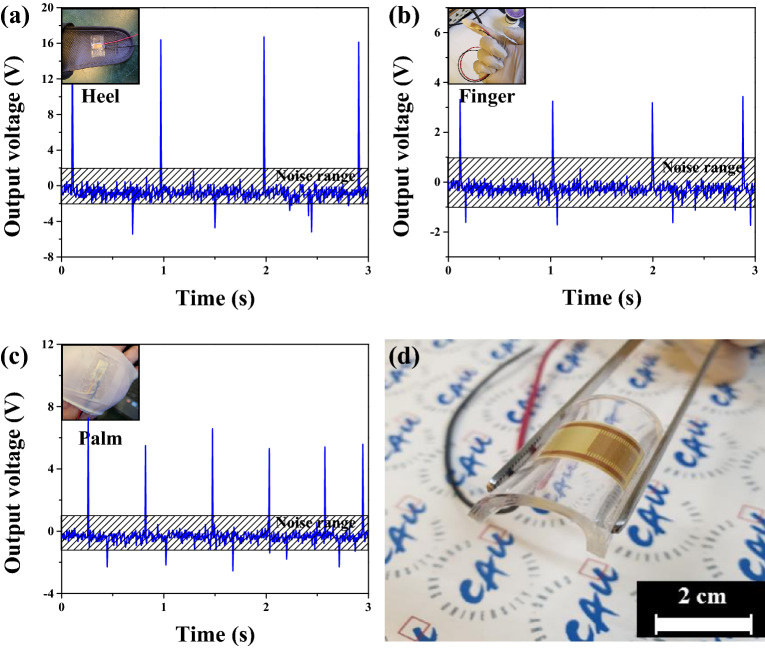
Output voltage when (**a**) pressed with the heel, (**b**) hit with the finger, and (**c**) when clapped with the palm the device and (**d**) the fabricated device.

Figure 9 shows the measured output voltage and the manufactured device under daily life conditions. The fabricated device was tested under stepping, tapping, and clapping conditions. The output voltages were 16.7 V and 3.4 V in the stepped and pressed conditions of the device. When the hand was clapping, an output voltage of up to 7.3 V could be obtained, and a random output voltage is measured. This result is due to the irregular impact applied. The measurement conditions were operated at 23 °C, 47% temperature and humidity conditions.

## Conclusions

In this study, N-rGO-doped P(VDF-TrFE)/BiScO_3_–PbTiO_3_-based composite nanofiber films were prepared using electrospinning. Conductive N-rGO was doped as a floating electrode material for the P(VDF-TrFE) polymer and the BiScO_3_–PbTiO_3_ polymer-ceramic composite. In addition, by generated output power, the maximum value of power density could be calculated used impedance matching. This composite-nanofiber-based energy harvester showed an improved output power owing to floating electrode effects. An interdigital electrode, which is an effective electrode structure for use in wearable device applications, was designed and tested. The generated output power was maximized by optimizing the fabrication process and the interdigital electrode design. The obtained open-circuit voltage, stored voltage, and generated output power were 12.4 V, 3.78 V, and 6.3 μW, respectively. As a result, floating electrodes in the composite nanofiber improved the output power and the effective dielectric permittivity.

## Data Availability

All data generated or analyzed during this study are included in this published article.
